# Changes in Oral Health, Oral Behaviours, and Oral Healthcare Utilisation Among Indian Migrants Living in the Netherlands

**DOI:** 10.1016/j.identj.2025.103946

**Published:** 2025-10-10

**Authors:** Amandeep Pabbla, Charles Agyemang, Irene Aartman, Denise Duijster

**Affiliations:** a Department of Oral Public Health, Academic Centre for Dentistry Amsterdam (ACTA), University of Amsterdam and VU University, Amsterdam, The Netherlands; b Department of Public and Occupational Health, Academic Medical Centre (AMC), University of Amsterdam, Amsterdam, The Netherlands

**Keywords:** Oral health behaviour, Oral health access, Immigrants, Asian Indians, Qualitative research, Good health and well-being

## Abstract

**Objectives:**

The aim of this study was to assess perceptions on the role of migration on oral health, oral health behaviours, and oral healthcare utilisation among Indian migrants living in the Netherlands.

**Design:**

Seven focus group discussions, with a total of 27 participants, were conducted among Indian migrants, using a semistructured interview guide. Interviews were transcribed and data were analysed using an open coding method, based on Andersen’s behavioural model. Using directed content analysis, emerging themes were organised under the main Andersen’s healthcare model.

**Results:**

The analysis resulted in six identified themes on changes perceived by Indian migrants in their oral health and oral health behaviours since migrating to the Netherlands, and in seven identified themes on changes perceived in their oral healthcare utilisation and identified barriers and facilitators with oral healthcare. Migrants reported shifts in product use, with some adopting Dutch oral hygiene routines while many also continued Ayurvedic practices. Positive dietary changes, such as reduced sugar consumption, were noted. Experiences with Dutch oral healthcare utilisation were mixed: some participants highlighted facilitators including practitioner honesty, fixed pricing, preventive orientation, and welcoming clinic environments, supported by dental insurance and recall systems. At the same time, barriers emerged, particularly related to limited communication, distrust in Dutch dentists, uncertainties about malpractice procedures, and challenges in navigating the referral system.

**Conclusion:**

This research highlights the complex factors associated with oral health behaviours and dental utilisation among Indian migrants in the Netherlands, emphasising the need for comprehensive consideration of cultural, socioeconomic, and environmental factors in future studies and interventions.

## Introduction

As of 2022, approximately 17.9 million Indian have migrated to different parts of the world.^1^ However, the process of migration is not as simple as physical relocation. Transitioning on multiple levels is generally overwhelming and can take a toll on health, including oral health, among migrants.^2^ Indians in particular carry a massive burden of oral diseases, with one third of global oral cancer cases being diagnosed among Indians.^3^ In addition, half of the Indian adult population suffers from some form of periodontal disease.^4^ Also, Indians generally have a lower rate of utilizing oral healthcare services due to a multitude of reasons, including financial and time constraints, low priority to dental problems, traditional beliefs and limited preventive and regular dental care.^5^

Dental literature indicates that after migrating to different countries, Indian migrants continue with the pattern of low use of dental services.^6^ As migrants, they express inability to speak the host language, lack of trust in foreign dentists as they may lack sensitivity or fail to understand the cultural differences which hinders clear communication between migrant patients and dental professionals. which were identified as additional barriers to dental service utilisation.^6^, ^7^, ^8^ However, studies on oral health among Indian migrants in Europe are sparse and far from exhaustive.^7^^,^^8^ Especially in the Netherlands, there has been a gradual increase in the number of Indian migrants in the last two decades. As of 2019, an estimated 58,460 Indians (exclusive of Surinamese Hindustanis) are living in the Netherlands.^9^ Hence, a better understanding of the oral health of Indian migrants will help to provide an easier access to oral healthcare utilisation in the Netherlands.

Andersen’s behavioural model is one of the most well-known models of healthcare utilisation,^10^ which states that ‘the people’s use of health services is a function of their predisposition to use services, factors which enable or impede use, and their need for care’. This model has been widely used in dental research to understand oral healthcare utilisation among specific groups, such as migrants,^11^^,^^12^ including refugees.^13^ Within this model, oral healthcare utilisation is determined by three main categories of factors: predisposing factors, enabling factors, and need factors. Predisposing factors predict the likelihood of people’s need for oral health services include population characteristics such as age, gender, and ethnicity. Studies in migrant groups have observed that males with low socioeconomic status (SES) had lower utilisation of dental services.^11^ Health beliefs related to oral health are considered equally important and have shown that migrants with higher cultural competence tend to be more satisfied with the dental utilisation in the host country, relative to those with lower scores.^14^ Enabling resources measure health-seeking behaviour and subsequent ability to use oral health services. Although studies on personal and family resources among migrants have found that financial constraints hinder dental service use,^15^ limited attention has been given to community and social relationships. Finally, the need component of the Andersen’s model considers how people view their own oral health in general and their experiences of symptoms and illness. Migrants often regard their oral health as poor, and yet their oral healthcare use varies considerably.^15^ Furthermore, personal oral health practices such as diet, oral health related self-care can also influence dental healthcare utilisation outcomes. Studies have shown that migrants tend to consume more sugar related food with lower brushing frequencies compared to the host population.^15^ This model also acknowledges the external environment (physical, political, and economic components) as important determinant for understanding the use of dental services. Level of integration,^16^ awareness of available infrastructure in the host country^7^ and effects of migration can impact utilisation of oral health services for migrants.

The primary aim of the study was to use Andersen’s behavioural model for healthcare utilisation to understand perceptions of the role of migration on oral health, oral health behaviours and oral healthcare utilisation among Indian residents living in the Netherlands. The secondary aim was to explore the facilitators and barriers perceived by the Indian migrants with the Dutch oral health care system compared to India. Given the aim of the present study was to gain a deeper understanding of perceptions on the role of migration and other factors associated with oral health outcomes among Indian migrants, comparisons with the host population were not within the scope of this research question. These have been reported elsewhere.^15^^,^^17^

## Materials and methods

### Study background and research design

This qualitative research is a part of a wider investigation, combining quantitative and qualitative research methods to explore perceptions of oral health, oral health status, oral health behaviours and oral healthcare utilisation among Indian migrants living in the Netherlands. For the project reported here, the focus was on the qualitative aspect by assessing the perceived role of migration on the oral health outcomes among Indian migrants only. This was done to gain a deeper understanding of their perceptions and needs regarding oral health, what enables them to maintain oral health, and how their experiences in the Netherlands have acted as facilitators or barriers in utilising oral healthcare services.

### Participant recruitment

Focus groups were conducted between November 2020 and 2021. In the previous study, researchers had asked participants (*n* = 147 Indian migrants), if they could contact them to invite them for a qualitative follow-up study.^17^ That study sample consisted of a random selection of Indian migrants who were 18 years and above, born in India and living in the Netherlands for at least five years in five major cities, namely: Amsterdam, including Amstelveen, Utrecht, Rotterdam, The Hague, and Eindhoven.^17^ The data was obtained from Central Bureau of Statistics, Netherlands (CBS), who drew a stratified random sample from the population registry of Dutch Municipalities (Basic Registration of Persons: BRP). For this qualitative study, all willing participants (*n* = 14 of the 147), who said yes to participation in the focus group discussions received an online Qualtrics link via email, that directed them to a short questionnaire. This questionnaire comprised of a consent form and information on their demographic characteristics such as age, gender, length of stay in the Netherlands, place of origin in India (name of state and city in India), highest level of education, and occupation in the Netherlands, language preference for the focus group, if any and time preference. This short online survey was available in English, Hindi, and Dutch. This information was requested to make focus groups more homogenous (Appendix Table 1), and the groups were categorised according to gender, to make them feel comfortable.

To include a broader range of perspectives (for Indian migrants who may not have been included via the quantitative survey), snowball sampling was also used. For this, information pamphlets (paper based and digital) with invitation requests for the Indians to participate in the group discussion were distributed and they had the contact details of the principal investigator (email and phone number). The heads/priests and community leaders of the various temples or worship places for Indians and Indian grocery stores in Amsterdam were also requested to distribute these pamphlets. Those Indians who thus contacted (*n* = 20), were requested to provide the above-mentioned information via Qualtrics or the principal investigator filled the details on the phone call.

### Approach to data collection

A semistructured open ended interview guide was developed based on Andersen’s Behavioural Model and pretested before using it for the focus group discussions. All necessary changes were incorporated, and the final interview guide was drafted after series of discussions between the team (Appendix Figure 1). The interview began with an open friendly question of introduction, warming up to their views on a healthy mouth, followed by their oral hygiene practices and diet and how migration influenced their perception and behaviours. The conversation shifted towards how they accessed oral healthcare, drawing from their experiences with dentists in both India and the Netherlands. They delved into the factors that either facilitated or hindered their dental visits, exploring the nuances of oral healthcare systems in each country. The discussion ended with a summary of the conversations and any take home message that stood out for them. These sessions lasted between 45 and 90 minutes.

### Setting and research team

As data were collected during COVID-19, two methods of conducting group discussions were adopted: online and in person. With no physical contact between the researchers and the participants, five were held online via zoom application (Zoom Inc.) and two group discussions took place in person at the place of worship (temple). The group discussions were conducted by the principal investigator (AP), who is of Indian origin and possesses experience in dental public health. The observer, a Master’s student in Dentistry at ACTA, Amsterdam, assisted with the project.

### Data recordings, and transcription

All interviews were conducted in either English, Hindi, or Punjabi before transcribing them into English. The principal investigator who is fluent in all three languages translated these interviews and documented them anonymously. During translation, the transcripts that were not in English, were read and reread. In the next step, these transcripts were translated into English by AP. The English was checked by a third person (English speaker), not related to the project. The translated documents were back translated into original transcripts by the principal investigator and were reread by Hindi and Punjabi teachers to ensure that the meaning was not lost in translation.^18^

### Codebook creation and data analysis

This project used both inductive and deductive coding methods to develop and describe the codebook. The deductive coding method was used to create an initial codebook development that would guide the analysis process. It was based on the research question, review of the relevant literature, and questionnaire that was developed for the quantitative phase of the project. The inductive coding method was also used so that any unexpected themes that may emerge during coding process would not be missed or overlooked (appendix table 2). This combination approach has been used in literature to answer the research questions in a holistic manner.^19^, ^20^, ^21^ Four basic components for codebook creation were used: the codes, code definition, code description, and an example text (appendix table 3).

Data were analysed using the software program Atlas.ti 22 (version 22.1.5.0). Directed content analysis was chosen as the method to analyse the data from focus groups. This form of analysis focuses on the systematic classification of data using coding to identify the key categories within it.^19^^,^^22^^,^^23^ First the transcripts were examined several times to get an overall impression of the data. Then initial open coding was done, and all codes were given a brief description with examples from the dataset. This was done independently by the principal investigator and the observer as a part of directed content analysis. The codes were then discussed, and minor modifications were made.^22^^,^^23^ All the codes were assessed, and similar codes were sorted under themes. These themes captured all important aspects of the Andersen’s behavioural model, including the external factors, population characteristics, oral health related behaviours and the oral healthcare utilisation.^10^ Finally, the themes were organised under the main model as a part of deductive coding (appendix table 4).

### Ethics

This study was approved by the Medical Ethics Review Board of the Medical Centre of the VU University Amsterdam (Reference number 2020.410).

## Results

### Participants

After conducting seven group discussions, a point of data saturation was reached as no new information was obtained. The responses in the last two groups were repetitive, leading to conclude the group discussions. The mean duration of the interviews was 53 minutes. In total, 27 Indian migrants participated in the seven interview sessions (eighteen males and nine females). Fourteen participants from the questionnaire survey and 13 out of 20 participants from snowball sampling contributed to the group discussions. Among the seven groups, four groups (group 1, 2, 3 and 4) consisted of Indian migrants working in the IT sector or holding various positions in business, all of whom had higher educational backgrounds and felt at ease engaging in the discussions in English. The remaining three groups (group 5, 6, 7), including the group of housewives, comprised individuals with lower levels of education, employed as construction workers, managing small grocery stores, or working in small scale catering business or tourism industry. Members of these groups preferred communicating in Hindi and Punjabi languages (Table 1).

**Table 1:** Composition of focus groups according to migration status.

Group	*N*	M:F ratio	Age group	Reason for migration	Education status	Occupation in the Netherlands	Time of residency in the Netherlands
1	3	3:0	20-30	Work	University degrees (masters)	In IT sector	≤5 y
2	3	3:0	35-45	Work	University degrees (masters)	In IT sector	5-10 y
3	3	2:1	35-45	Family, work	University degrees (graduates or masters)	In business sector	5 y
4	4	4:0	45-55	Work	University degrees (graduates or masters)	In IT, business sector	5-20 y
5	5	5:0	30-50	Family, work	High school pass out	Construction workers, small shopkeepers	10-30 y
6	4	1:3	30-50	Family	High school pass out	In small scale tourism, recreation, catering sector	15-30 y
7	5	0:5	30-50	Marriage	High school pass out	Housewife	10-30 y
Total:	27						

M:F, male: female ratio; N, number of participants per group.

### Themes

**Fig. 1:**
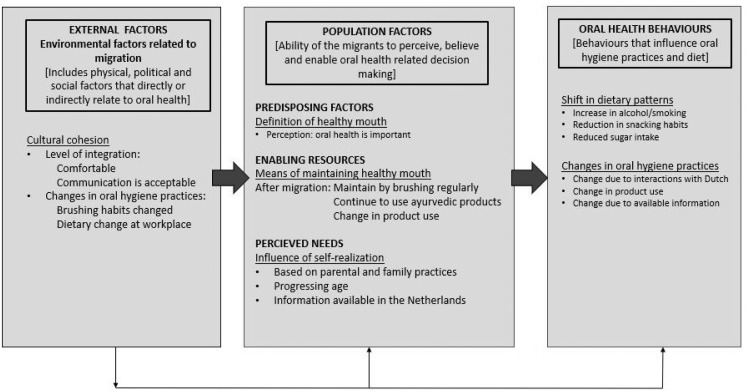
Model 1 summarizing the results on the changes perceived by the Indian migrants in their oral health and oral health behaviours since migrating to the Netherlands.

**Fig. 2:**
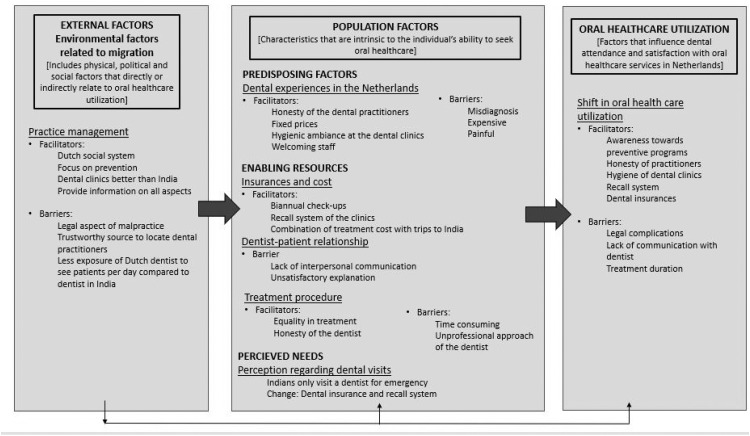
Model 2 summarizing the results of the changes perceived by the Indian migrants in their oral healthcare utilisation and identified barriers and facilitators with oral healthcare between India and the Netherlands.

The analysis resulted in a number of themes which were organised based on the Andersen model (Fig. 1, Fig. 2): (1) Changes perceived by the Indian migrants in their oral health and oral health behaviours since migrating to the Netherlands, with six themes and (2) Changes perceived by the Indian migrants in their oral healthcare utilisation and identified barriers and facilitators with oral healthcare between India and the Netherlands, with seven themes. These models are not mutually exclusive, although for clear description, each model is constructed with themes that are consistent with the domains of the Andersen’s model (predisposing, enabling and need factors).

### Changes perceived by the Indian migrants in their oral health and oral health behaviours since migrating to the Netherlands

The themes and related quotes can be seen in Table 2 (and Figure 1). These are briefly outlined below.

**Table 2:** Summary of categories, themes and quotes related to changes perceived by the Indian migrants in their oral health and oral health behaviours since migrating to the Netherlands.

Category	Themes	Relevant quotes by respondents
Individual factors [Predisposing factors]	Definition of a healthy mouth	a) ‘I think mouth health means my teeth should stay strong and healthy so that I have no problem in eating and drinking’ FGD-21/11/21 (Worker, males in person, GROUP-5) ‘Shiny white teeth' FGD-29/11/21 (IT sector, mixed online, GROUP-3) b) ‘I think…like we say in India "If teeth go, the taste goes too!". Hahahaha…So, teeth are very important and we need to be aware of their importance’ FGD-27/11/21 (Workers, mixed group online, GROUP-6) c) ‘Taking care of the mouth is very important. We do go to the doctor regularly’ FGD-29/11/21 (IT sector, mixed online, GROUP-3)
[Enabling resources]	Means of maintaining a healthy mouth *(Ayurvedic options)*	d) ‘Yeah, I, I think I brush twice’ FGD-29/11/21 (IT sector, mixed online, GROUP-3) e) ‘We mostly brush our teeth, once at least…maybe twice also’ FGD-21/11/21 (Housewives, females in person, GROUP-7) f) ‘I usually clean my teeth morning and evening…yea, everyday brushing’ FGD-21/11/21 (Worker, males in person, GROUP-5) g) ‘the products maybe have changed like the toothpaste or, or anything…but there has been no change, routine wise’ FGD-29/11/21 (IT sector, mixed online, GROUP-3)
		h) ‘For example, for the teeth related thing, mostly we use the Ayurvedic related products and paste…we could see the benefits, rather than going for chemical-based things. So, we always trust in that. And even after coming here also. I tried the Ayurvedic stuff…the tooth paste basically is made of some tooth powder, so yeah, the approach was basically the same.’ FGD-14/09/21-(IT sector, male online, GROUP-2)
		i) ‘yes, mostly in India, we used twigs only. Or else, coal and oil. Or we had only one Colgate (toothpaste). Its name was only Colgate…when paste used to be over in India, we used to put salt on our palm, dad used to say, come get some salt. That was it.’ FGD-21/11/21 (Worker, males in person, GROUP-5) j) ‘But here, mostly we brush our teeth only with brush and toothpaste. And also use mondwater (mouthwash) for rinsing’ FGD-27/11/21 (Workers, mixed group online, GROUP-6)
[Perceived needs]	Influence of self-realization *(parental/ family influence)* *(Age related improvement)* *(Information in the Netherlands)*	k) ‘I see my life a bit different. I mean, we are all from India, but typically when we got married, I saw her (wife) dental habits far better than mine. So…dependent on the family and the upbringing…maybe relates to the family culture. I mean, she used to brush like twice…and then I'm have a Eureka moment…she forced me also to get into those good habits, saying it helps’ FGD-14/09/21 (IT sector, males online, GROUP-2)
		l) ‘I mean, it's difficult to say, let's say today, if I was in India, I would not be doing, I would not be brushing my teeth twice. Like I would have a bad oral hygiene because I don't know. For me it, of course it improved now after coming to Netherlands. But I think it has more…you should, how to maintain oral hygiene…so for me, it's more related to age’ FGD-14/09/21 (IT sector, males online, GROUP-2)
		m) ‘I would say that, you know, after coming to Netherlands, uh, at least I have to some extent started, you know, giving more care to my teeth than I was in India, for sure… Yeah,…I mean, for me, it was really, the awareness was created by the infrastructure in place, in Netherlands…information on infrastructure available.’ FGD 5/11/20 (IT sector, males online, GROUP-1)
Contextual factors	Cultural cohesion	n) ‘Well, I, I I've done that, that integration course, and I feel quite comfortable. But I cannot also claim to be 100% integrated or kind of Dutch. But I ask myself that, I need to keep my own identity as well. So, you know, as long as I can mix with others…then I'm making progress. I can make a peace with that…And for that very reason, I've enjoyed my stay here quite a bit’ FGD-14/09/21 (IT sector, males online, GROUP-2) o) ‘‘And I'm also not faced any situation where they had to really chat with me in Dutch. So, if I just tell them that I don't understand that they, they just quickly switch to English.’’ FGD-29/11/21 (IT sector, mixed online, GROUP-3) p) ‘I'm in Amstelveen Amsterdam area, where it is far more international’ FGD- 14/09/21 (IT sector, males online, GROUP-2) q) ‘‘Yea, but that is not all that bad. I mean, we women have to do all things by ourselves here, so language is not all that big an issue, right.’’ FGD-21/11/21 (Housewife, females in person, GROUP-7)
		r) ‘Diet, I think…after coming here seeing the Dutch culture and the way they eat and even the exercise and taking care of your body and those kinds of things. That has really improved, I would say…has a positive impact after coming here I would say’ FGD-14/09/21 (IT sector, males online, GROUP-2) s) ‘Since moving to the Netherlands, for me it has improved (oral health behaviours)…mainly the s brush before the night, it's good for oral hygiene. It's not a chore’ FGD-14/09/21 (IT sector, males online, GROUP-2) t) ‘For me, the, the, not the infrastructure, but the people…have been the influence, like their habits and that has been in reinforced into them from the childhood. It's really the generation who follows the habit of a good oral hygiene. They, they made me change’ FGD-14/09/21 (IT sector, male online, GROUP-2)
Oral health outcome Oral health behaviours	Shift in diet *(Smoking and alcohol*) *(Changes in food)* *(Sugar intake)*	u) ‘I think smoking and, uh, drinking, they increased when I came to Europe. And when I go back to India, also, that's a culture aspect that smoking and drinking is not considered, uh, a good virtue in the Indian culture, but here it's completely normal’ FGD-29/11/21 (IT sector, mixed online, GROUP-3)
		v) ‘If only the situation is a bit different, maybe it's the surroundings or the environment that influences…In India…I'll say vada pao (Indian snack) is easily accessible. And when we just step out of an office, okay, let's grab some snacks or have some fast food…In India, it's easy to get a fast food and really tempting…But given the situation here (in the Netherlands), maybe more correlated to the professional life and the scenario in Netherlands, I don't think we can grab such fast food so often’ FGD-14/09/21 (IT sector, males online, GROUP-2)
		w) ‘For me, the two big changes I noticed, like I stopped drinking tea with milk mostly…I would have never done it in India. I was in India. I would always drink black tea with milk and sugar always. And probably that's because it's always available…But here it's not available. I have to make it myself. So, I moved to that, just hot water and a pack, a sack of green tea or chamomile. And that's, sugarless, that's without milk. I think it's better for appetite. So that was a change. I'm happy with it’ FGD-14/09/21 (IT sector, male online, GROUP-2)
	Changes in oral hygiene practices *(Group 1-4)* *(Group 5-7)*	x) ‘But it changed more because of my colleagues, for instance, like the friends I have here (the Netherlands). So I was on a trip to Belgium with my Dutch colleagues…So we were like at the night, when we came back from a nightclub and it was like, 2:00 AM, we had to go to bed…everybody take, takes out their brushes and they start brushing. And I was so shocked. I was like, 2:00 AM. People are drunk. And they, they still remember, they have to brush and then go to sleep. And I didn't…So that was kind of a shocker’ FGD-14/09/21 (IT sector, male online, GROUP-2) y) ‘The way I approach (dental problems) is the same, how I approach in India, as well as here. For example, for the teeth related thing, mostly we use the Ayurvedic related products…We could even able to see the benefits…So we always trust in that. And even after coming here also, I, I faced one kind of problem in my teeth. And I tried the Ayurvedic stuff…so yeah, the approach was basically the same’ FGD-14/09/21 (IT sector, males online, GROUP-2)
		z) ‘In India, it (brushing) was like a routine, while getting ready for school, we would brush in the morning only. Not in the evening. But since we moved here…because here we go to the dentist and they advise us, right…to brush twice, otherwise we might get pain, or something…So here the habit has changed’ FGD-21/11/21 (Housewife, females in person, GROUP-7)

#### Definition of a healthy mouth

A healthy mouth was observed as a predisposing factor. Indian migrants defined a healthy mouth as the ability to bite, taste and eat everything, having stronger and shiny white teeth (a). They also perceived their mouth to be important, hence keeping the mouth clean and healthy by either brushing or visiting the dentist regularly was considered mandatory (b, c).

#### Means of maintaining a healthy mouth

Various methods that Indians used to keep their mouth clean were observed as an enabler for maintaining a healthy mouth. Indians brushed their teeth at least once daily (in the morning) (d, e, f). After migrating to the Netherlands, Indian migrants from group 1 to 4 (higher educated) felt no change, since they brushed their teeth in India as well as in the Netherlands (g). In addition, Indians continued using Ayurvedic products for mouth cleaning in the Netherlands, because of their trust in them (h). However, Indian migrants in group 5 to 7 (lower educated), felt a change as they switched from commonly using twigs (branches of tree) and salt as a tooth cleaning powder in India, to toothbrushes and toothpastes (i, j).

#### Influence of self-realisation

Indians viewed the decisions on lifestyle, including oral health-related choices, as perceived needs. They identified needs as a matter of awareness towards surrounding environment and influence of migration. This included their parental and family practices related to hygiene maintenance (k). Also, increased awareness to maintain oral health was perceived as a result of progressing age (l), or due to the information they received in the Netherlands (m) on adapting to healthier oral hygiene choices.

#### Cultural cohesion

Apart from the mentioned population characteristics, external factors, such as level of integration was also assessed among Indian migrants. Indians predominantly engaged with Dutch locals beyond their families, feeling at ease with their level of integration (n). Those in groups 1 to 4 interacted with their Dutch friends in English (o, p), whereas individuals in groups 5 to 7 learned to communicate in Dutch (q). Regardless of the language used, many Indians comfortably embraced Dutch oral health practices, such as night time brushing and dietary changes in the workplace (r, s, t).

#### Shift in diet

Due to the changes in perception of oral health and oral health behaviours, a shift in diet was observed. Indian migrants felt an increase in their consumption of alcohol and smoking. As one respondent said, ‘habits are the opportunities to celebrate’. This was attributed to change in social norms, lifestyle change in the Netherlands and influence of peers (u). For changes in foods, Indians expressed a reduction in their frequency of snacking habits and observed they had shifted to healthier foods in the Netherlands (v). It was generally agreed that lack of availability of tempting Indian snacks, lesser time to prepare Indian food, healthier options available in the Netherlands and no snacking at professional work environment, was ‘blessing in disguise’. Furthermore, Indian migrants also indicated a positive shift towards lower consumption of sugar, which was more environmentally influenced (v, w).

#### Change in oral hygiene practices

The interviewees noted a shift in their oral hygiene routines after relocating to the Netherlands. Indians in groups 1 to 4 began brushing their teeth twice daily, a habit they adopted primarily through interactions with the Dutch and by observing the oral hygiene practices of their international peers (x). Despite this change, Indian migrants maintained their use of Ayurvedic products due to their enduring trust in these items (y). Notably, individuals in groups 5 to 7 experienced changes in their product preferences and began consulting dentists for expert guidance on enhancing their oral hygiene practices (z).

### Changes perceived by the Indian migrants in their oral healthcare utilisation and identified barriers and facilitators with oral healthcare between India and the Netherlands

The themes and related quotes can be seen in Table 3 and Figure 2. These are briefly outlined below.

**Table 3:** Summary of categories, themes and quotes related to the changes perceived by the Indian migrants in their oral healthcare utilisation and identified barriers and facilitators with oral healthcare between India and the Netherlands.

Category	Theme	Relevant quotes by respondents
Individual factors [Predisposing factors]	Dental experiences in the Netherlands *(Group 5-7)* *(Group 1-4)*	A. ‘Better than in India. I am not saying doctors in India are less educated, but there it is like shopping. For example, in India, you can choose to buy a cake for 10, 20, 50 or 100 Euros, you get to choose. So obviously if you choose for less price, your cake will rot soon. Here we have no price list here (Netherlands). Same price, any person. He went, same treatment, same price. For me also, the same. Here they do not say different things to different people. It is much better here’ FGD-21/11/21 (Worker, males in person, GROUP-5) B. ‘Here (Netherlands) they spread a paper with things on it, treat you and immediately wrap up everything in that paper and…prepare another for the next patient. But in India, they use clothes, and bad dirty clothes…Hahaha…really, and do treatment just like that’ FGD-21/11/21 (Housewife, females in person, GROUP-7)
		C. ‘Like I said, I lost two teeth in Netherlands…The extract was done and it was stitched up, it was still bleeding and I waited for four hours, five, still kept on bleeding and then I had to call, go to the emergency, but they referred to me to a doctor at 1:00 AM in the morning…then I went to the doctor and he did quote that; your stitch has not been done very well. And you've been swallow your own blood for the last six hours. So I didn't have good experience’ FGD-14/09/21 (IT sector, males online, GROUP-2)
[Enabling resources]	Insurances and costs *(Group 1-4)*	D. ‘And in the first two, three (y), I, I, I was not taking dental insurance, but then it came to the company…I had the insurance, but I was not taking it. But once I had these incidents (dental problems) that I had to go to dentist because of critical situation, then I realised, oh, this much it's all covered in, in the, the insurance. So for me initially, the perception was the cost, but then I realised, just take the insurance, which the company gives for free anyway. It, so cost is not the issue. It's, it's more my tendency to go there’ FGD-14/09/21 (IT sector, males online, GROUP-2) E. ‘So when I first went to the dentist over here (the Netherlands), it was not due to any problem I had. It was a, you know, when, when you do your medical insurance here, dental policy is part of the package as an option. And then you, you kind of try to figure out, and then you say… you are allowed to go to a dentist twice a (y), just for checkups. I said, ok, lets go!’ FGD-14/09/21 (IT sector, males online, GROUP-2) F. ‘an example of friends…Indian origin Dutch citizen. They have been here for the last 15, 16 (y) now…they all get their dental, you know, procedures done in India because of the fact that 800 euros, you can get a flight ticket back home. Yeah. Yeah. So it's like, you know, you can combine your travel and look, go and see your families back home…Right. So yeah, in 800 euros, you can go with your entire family, like a vacation to India, get your dental also done, come back. So it's so…why, why am I spending four or five grand here? I can just fly back home and, you know, get it done. Yeah’ FGD 5/11/20 (IT sector, males online, GROUP-1) G. ‘And so for me now…what I have planned…if I'm able to get 500 euros of imbursement, I would at least get that much of treatment from Netherlands. More than that, I will just wait until I go to India. So if I…manage to get everything I can get from, in insurance here. The rest time we do in India’ FGD-14/09/21 (IT sector, males online, GROUP-2)
	Dentist-patient relationship	H. ‘Information here is better. But treatment wise…For example, I went to the dentist here and he said you are getting old and that is why. I mean I am not yet 50 and…still he just declared that my problem with teeth is age related. So I didn’t like it. You know when you meet someone and you know, they don’t have to talk, talk, but you get a feeling…like how they handle you, that is not good here’ FGD-27/11/21 (Workers, mixed group online, GROUP-6) I. ‘It's, it would have rather not changed because here, I think in Netherlands, Dutch dentists, they explain less stuff. So I don't really trust their advice because they are very, they don't elaborate their advice. In India they would really elaborate their advice. So I already feel, they are saying it for my good’ FGD-14/09/21 (IT sector, males online, GROUP-2) J. ‘Earlier I had a white dentist, but for the past 2 (y) I have started going to an Indian dentist. I find that easy. Because he is ours. He knows Indian people better, I think. He advises us better’ FGD-27/11/21 (Workers, mixed group online, GROUP-6)
	Treatment procedures *(compare India and the Netherlands)*	K. ‘it takes them (dentist in Netherlands) time, also the treatment, or if they, they give me a plan, it's quite stretched out. Like, so the availability, like for me, it always feels if I have to get it done here, it'll take so long’ FGD-14/09/21 (IT sector, males online, GROUP-2) L. ‘The way she (dentist in the Netherlands) told me that, okay, this has to be done (root canal treatment). This has to be, then this is the, she did not properly explain the repercussions that, okay…And I mean, if I, I think if as a, as a patient or as a client, if I have no pain whatsoever before going there and she does a job and then as it starts paining, like anything, and I, I can't even sleep then I, I don't need that thing. Right. Yeah’. FGD-29/11/21 (IT sector, mixed online, GROUP-3). M. ‘They (Dentist) also take care while giving injections. Ask you 10 times, is there any pain, are you ok. In India, they just inject and say, this much pain is bound to happen’ FGD-21/11/21 (Worker, males in person, GROUP-5)
[Perceived needs]	Perception regarding dental visits *(in India)* *(change in Netherlands)*	N. ‘Well in, in India as, as, as you and others probably know, you generally don't go to a dentist unless you have a problem, unless you have an issue…And you assume that dentally speaking, you are fine. So, so that, that was my personal situation in India.’ FGD-14/09/21 (IT sector, males online, GROUP-2) O. ‘It is also in our insurance that we go do a dentist and if you don’t go then mostly they call you every three months or six months as a reminder.’ FGD-21/11/21 (Worker, males in person, GROUP-5)
Contextual factors	Practice management *(Dutch social system)* *(focus on prevention)* *(dental clinics)* *(barriers)*	P. ‘The difference is, maybe the labour laws, right. Here, a person can go on a stress leave for a mon paid or on a medical leave sickness-leave for three months because health is priority. In India, if you have our work contracts. And if we go to three months, leave, that's set with our jobs’ FGD-14/09/21 (IT sector, males online, GROUP-2) Q. ‘You know, I said like, uh, when the infrastructure provides you from going to, to go to a dentist and why not, and, and by the way, my children were in the international school here in Amsterdam. And, the international school itself had a program for small children to be part of the dental program. So I saw them, you know, go like every once in three months or, or actually the dentist would come to the school and then they would get their mouth checked. That also created the awareness. And, yeah, so I'd say, why should I be left out?’ FGD-14/09/21 (IT sector, males online, GROUP-2) R. ‘Doctors (in India) are not very good in their dealing. Like in some dental clinics, it is not very clean…like…we can see that their gloves, that can be dirty or with blood…you know. They start seeing patients like that…hygiene…here (Netherlands) the dentist are so careful and give so much importance to hygiene, right.’ FGD-21/11/21 (Housewife, females in person, GROUP-7) S. ‘They tell us based on your budget, what insurance to get, what treatment, what type of cleaning. I go after 3 months for cleaning and 2 times a year for control (with the dentist)’FGD-21/11/21 (Worker, males in person, GROUP5) T. ‘so I think that would also be one of the biggest barriers for me that if something goes wrong, what next. Medical, I know that there are processes set up, procedures, set up, everything is fine…there, there is an accountability system in place for medical specialist treatment…but with dentist you're unaware of it. Yeah. So there is a lot of lack of information. If something goes wrong, what next, if the dentist is not able to perform his job…What next..?” FGD 5/11/20 (IT sector, males online, GROUP-1) U. ‘I'm new here…I don't know how to select one (dentist). That is the biggest thing. Like, uh, how do you find a good one? Because there is no, there is no list. Like these are the good ones. No…So I don't want to just land up on a doctor's lab and I don't know, cause over in India you can get recommendations with a lot of your friends and other families, but over here it's difficult…it was not really, uh, easy to find a good one’ FGD 5/11/20 (IT sector, males online, GROUP-1) V. ‘In my view, uh, what I have come to understand is that the, the, the amount of people that the professionals (medical and dental) see over here is far, far less than what any medical professional in India would encounter. So a person, a doctor (or dentist) in India on an average might attend to anywhere between 50 to hundred patients, but here they, they have appointments hardly for, uh, you know, 10 people. So that's, that also makes, uh, a big difference. I think’ FGD 5/11/20 (IT sector, males online, GROUP-1)
Oral health outcome	Changes in oral healthcare utilisation *(facilitators)* *(barriers)*	W. ‘Yeah, for me, certain degree of improvement for sure… And at least the dental hygienist, give all the right advices, to make sure I stay healthy. And those reminders of course, make sure that I visit, far off and, compared to what I did in India’ FGD-14/09/21 (IT sector, males online, GROUP-2) X. ‘So knowledge wise as well, they feel less because they don't explain much. Probably, maybe I don't speak Dutch. So we, we always talk in English. Maybe that could be the reason, but I don't know’ FGD-14/09/21 (IT sector, males online, GROUP-2) Y. ‘I went to a reception for one of the dental places I could get myself for the register and the receptionist could not understand English. And then I tried to explain and fight 10 minutes in broken English, didn't work out and I had to walk out, but I'd say that's more of a problem from my side, knowing that, of course, a local knowing a local language’ FGD-14/09/21 (IT sector, males online, GROUP-2)

#### Dental experiences in the Netherlands

The overall experience of Indian migrants with the Dutch dental care was seen as predisposing factor. Indians agreed that visiting a dentist in India was mostly for emergency reasons such as pain or swelling. Since their migration, many Indians from group 5 to 7 visited a Dutch dentist and talked about positive dental experiences in the Netherlands. Common facilitators mentioned were honesty among the dentist, fixed prices, good hygiene in dental clinics and welcoming staff in the Netherlands (A, B). Furthermore, they mentioned that now they live in the Netherlands, so this is their ‘home’, and this is where they want to go for dental visits While Indian migrants from group 1 to 4 only visited a Dutch dentist for emergency purposes and described their dental experience in the Netherlands as ‘bad’. They talked about how their dental problems were misdiagnosed and became expensive and painful for them (C). Hence, their preference was towards dentists in India.

#### Insurance and costs

Despite the compulsory health insurance, Indians, especially in groups 5 to 7, opted to buy additional dental insurance. The prevailing consensus among Indians was that insurance, whether for basic medical needs or supplementary dental care, served as an enabler. Basic medical insurance, a standard for all, offered the advantage of biannual dental check-ups, motivating Indians to visit a dental clinic (D, E). Indians strategically combined their dental appointments with trips to India, viewing it as an opportunity for more economical treatments alongside family reunions (F). Individuals with dental insurance utilised their coverage in the Netherlands, deferring uninsured treatments until their visits to India, where affordable dental care coincided with family gatherings (G).

#### Dentist-patient relationship

This theme was identified as an additional facilitative factor, characterised by interpersonal relationships, which encompassed communication with the dentist. Despite the built-in recall system associated with dental insurance, which was viewed as a facilitator for dental visits, the absence of interpersonal communication with the dentist was generally regarded as a hindrance. Indians felt that the dentist did not invest enough time in them to develop any personal communication. As a result, Indians perceived the explanations related to treatment as unsatisfactory, which ultimately translated to reduced trust in the dentist (H, I). Therefore, Indians preferred to search for a dentist of Indian origin for their dental treatment in the Netherlands (J).

#### Treatment procedure

A frequent point discussed was the comparison of the treatment procedures between the two countries (India and the Netherlands), which was also an enabling resource. Despite the positives of insurances, Indian migrants preferred the treatment in India because they felt that in the Netherlands, treatments are usually time consuming and ‘unnecessarily’ prolonged (K, L). Indians from group 5 to 7 preferred the treatment in the Netherlands because of equality in treatment, and honesty of the dentist (M) as mentioned previously and environmental influences, discussed below.

#### Perception regarding dental visits

Indians expressed that they had never visited a dentist in India until they had a dental problem or in an emergency. Hence perceived needs to visit a dentist did not involve routine/regular dental visits as this is not a norm in Indian society (N). This perception did not change for some even after moving to the Netherlands. However, Indians with dental insurance noticed a change in their frequency of dental visits, which they attributed to the recall system of the clinics in the Netherlands (O).

#### Practice management

From the environmental context, certain key factors emerged as facilitators for oral healthcare utilisation. Firstly, Indians praised the benefits of the Dutch social system, where priority is given to the health of the employee (P). Also, Indian migrants were impressed with how the focus of dental care in the Netherlands is on prevention, be it via the school programs of their children or their regular visits to the dental hygienist (Q). Furthermore, Indians compared the Dutch dental clinics with India and agreed that dental clinics in the Netherlands were far more welcoming and hygienic (R). In addition, Indians liked the system of Dutch dental clinics providing them with relevant information on treatments, appointments, and insurances (S). However, Indian migrants from group 1 to 4 considered Dutch oral healthcare as ‘average’ and only liked the dental hygiene aspect of it. They also felt unsure on what to do if the dental treatment did not go well in the Netherlands and considered this as a barrier (T). In addition, they expressed a lack of trustworthy source to locate a good dental practice in the Netherlands (U). They also compared the dentist population ratio between the two countries (India and the Netherlands) and felt the exposure of the dentist in treating the number of patients per day is lesser in the Netherlands compared to the dentist in India (V).

#### Changes in oral healthcare utilisation

Oral healthcare utilisation for Indians has changed since they moved to the Netherlands. The major facilitators mentioned were insurances, exposure to preventive programs and recall system of the dental clinics, which had motivated them for routine dental visits (X). As for the barriers, Indians expressed their communication with the dental team, including the dentist as unsatisfactory. This barrier was partially explained as lack of trust. Environmentally, dissatisfaction in communication was attributed to either their poor knowledge of Dutch language or lower cultural sensitivity among Dutch dentists (Y, Z).

## Discussion

The main findings of the study were that Indian migrants perceived their oral health as important and maintained it through regular brushing. After migration, their resources shifted, with changes in product usage, although many continued with Ayurvedic products. Perceived needs also evolved due to factors such as progressing age and available information in the Netherlands. External influences, especially integration levels, shaped their oral health behaviours, as they comfortably adopted Dutch oral hygiene practices. Dietary habits also shifted, with reduced sugar consumption and snacking seen positively, while increase in smoking and alcohol consumption was reported. In terms of dental utilisation, Indian migrants initially did not prioritise routine dental visits in India. However, upon migration, those who perceived positive experiences in the Netherlands attributed their willingness for dental visits to factors like the honesty of practitioners, fixed prices, hygienic clinic ambiance, and welcoming staff. Dental insurance emerged as a significant facilitator, enabling biannual check-ups, and often synchronised with their visits to India. Environmental aspects such as labour laws, clinic atmosphere, recall systems, and preventive programs also encouraged dental visits in the Netherlands. Conversely, barriers included distrust in Dutch dentists, uncertainties about malpractice laws, limited exposure of Dutch dentists to a high patient load, and a lack of a reliable referral system.

Focus group interviews showed that Indian participants regarded oral health an essential component of overall wellbeing. This supports the findings of Batra et al. where Indian migrants had a good knowledge of their oral health and were conscious of oral health being a part of holistic health, with the needs to maintaining oral hygiene.^6^ In this study, Indian migrants maintained their oral hygiene, using toothbrushes, dentifrices, or additional Ayurvedic products. This finding is in accordance with MacEntee et al.,^24^ who also observed that Indian migrants in the Canada combined Ayurvedic products, including home remedies with toothbrushing. This seems only natural as Ayurvedic products form a traditional as well as scientific basis of maintaining oral hygiene in India.^25^^,^^26^

Oral health beliefs and practices are also influenced by changes in the immediate surroundings of an individual. In the present focus groups, Indians experienced change in their oral hygiene practices as they adopted healthier oral health behaviours, which were partly influenced by their interactions with the Dutch. These findings are in contrast to other studies which suggest that migrants with lower education and employed as low-skilled tend to transition towards a higher-sugar diet in the host country.^8^ In addition, factors such as adaptability (integration), mother’s language proficiency and length of residency are shown to be associated with changes in oral health behaviours among migrants.^16^ However, since studies on the role of migration on oral health behaviours among Indian migrants is sparse, more research is needed to deepen our understanding on this topic. Also, research on oral beliefs and practices of Indian migrants may help identify unique perspectives and potentially inform culturally sensitive dental care strategies.

In contrast to the Netherlands, routine dental visits are not the norm for everyone in India.^27^ Hence, Indian migrants perceived the need to visit a dentist only in emergency dental problems such as pain or swelling in the mouth. Recent systematic research has also revealed that the overall dental utilisation in India is around 24%.^28^ However, findings from these focus groups suggest that Indian from group 5 to 7 visited a dentist regularly, partly due to dental insurance coverage. These participants, who generally had lower education and lower paying jobs, described their dental experiences in the Netherlands as positive and were satisfied with the treatment procedures offered. Studies in rural parts of India have shown that overall patients’ satisfaction level with the dentist is satisfactory since they are often unaware of their dental problems and rely heavily on the expertise and advice of the dentist.^29^^,^^30^ External factors, such as priority given to the employee’s health, friendly and hygienic ambiance of dental practices, standardised rates of treatment and recall systems, further encouraged their preference towards utilising dental care in the Netherlands, rather than relying solely on trips to India.

At the same time, our findings revealed contrasting views among participants with higher education and professional backgrounds. These migrants were less accepting of Dutch oral healthcare, which may be explained by prior experiences with private care in India and the higher expectations they brought regarding efficiency, communication, and treatment standards. In contrast, lower educated migrants valued aspects such as fixed pricing, hygiene, and the preventive focus of Dutch oral healthcare, which they may perceive as clear improvements compared to their experiences in India. This contrast highlights how prior frames of reference and expectations shape perceptions of oral healthcare systems after migration.^14^

Despite these facilitators, Indian migrants regarded their communication with the dentist in the Netherlands as unsatisfactory, either due to their inability to speak Dutch or because of cultural differences. This resonates with the findings of other researchers where distrust in dental care,^31^ limited language proficiency^32^ and poor interpersonal relationship^33^ were highlighted as barriers by migrants. Studies have shown that cultural competency is instrumental in encouraging greater sensitivity and understanding of patients’ needs, which consequently impacts the utilisation of oral healthcare in the host country.^34^ This is evidenced by practices such as simplifying dental information, treatment, and preventive measures using multiple languages, incorporating pictorial representations, and including culturally diverse individuals and food examples in informational charts and diagrams.^34^ Furthermore, Indians also felt poorly informed on the laws related to misdiagnosis or negligence in treatment carried out. Such uncertainties consequently led to lower confidence in the dentist and hence limited their use of dental services in the Netherlands. Since dental workforce is at the heart of oral healthcare, they play a crucial role in not only emergency care that Indians are more used to, but also preventive care, that can encourage the practice of routine dental visits among them.^35^ These findings emphasize the need for cultural competence and interpersonal approach, acknowledging cultural nuances and building trust through genuine connections. This approach can foster patient satisfaction, adherence to treatment plans, and overall positive oral health outcomes within migrant communities. Therefore, developing policies that encourage and support dental practitioners to provide culturally sensitive oral healthcare services to Indian migrants could potentially enhance their dental visits. Additionally, investments in public awareness campaigns regarding available preventive programs and the importance of regular dental check-ups may empower migrants to make informed decisions about their oral health.

### Strengths and limitations

Indian migrant population in the Netherlands represents a diverse group in terms of culture, language, and lifestyle practices. Focusing on the study of a specific migrant group provides valuable insights into understanding the intricate influences of migration on oral health behaviours and utilisation patterns. By focusing on a particular migrant community, this study was able to delve deeply into the unique cultural, social, and economic factors that impact oral health within the context of Indians. This targeted approach allows for a nuanced analysis of how migration-related stressors, acculturation challenges, and access to healthcare services interact to shape oral health behaviours. However, it is essential to recognize the limitations of such a focused study. While the findings offer in-depth understanding within one group, extrapolating these results to other migrant communities must be done cautiously. Different migrant groups often face diverse challenges, ranging from language barriers to varying levels of social support, which can significantly influence oral health behaviours.^36^ Therefore, while specific insights can be gained from a singular migrant group, broader conclusions about migration’s impact on oral health should consider the diversity inherent in various migrant communities.

Furthermore, most focus groups were conducted online; hence participants' body language (gestures, eye contact) was not subject to observation. Secondly, due to participants' environment (eg, home, office), distraction-free discussion among focus group members may not have been possible.^37^ Another limitation of this research is the potential lack of comprehensive representation within the studied focus groups. While efforts were made to ensure that participants were included from different education level, occupational background and genders, the findings should be interpreted with caution. This representation might not have captured the full spectrum of experiences within the Indian population, especially considering the vast socioeconomic, cultural, and regional diversity that exists within India. Also, no causal conclusions can be drawn about the role of migration on the perception of oral health, oral health behaviours, and oral healthcare utilisation among Indian migrants living in the Netherlands in this cross-sectional qualitative study.

## Conclusion

In conclusion, this research significantly contributes to understanding the intricate dynamics of oral health behaviours and utilisation patterns among Indian migrants in the Netherlands. By identifying the factors that are associated with both oral health practices and visits to dental professionals, this study provides valuable insights with broader implications. Furthermore, these insights highlight the necessity of accessible dental insurance options, clear communication channels, and robust referral systems. Policymakers can leverage this understanding to design initiatives specifically tailored to address these challenges, thereby ensuring equitable access to dental care services for migrant populations. Ultimately, these research findings have a potential to initiate a positive cycle of understanding, integration, and improved oral healthcare outcomes, benefiting both the migrant communities and the host country.

## Conflict of interest

The authors declare that they have no known competing financial interests or personal relationships that could have appeared to influence the work reported in this paper.

## Appendix Supplementary materials

Image, application 1

## Footnotes

Supplementary material associated with this article can be found in the online version at doi:10.1016/j.identj.2025.103946.
