# Supplementary material for: Changes in Oral Health, Oral Behaviours, and Oral Healthcare Utilisation Among Indian Migrants Living in the Netherlands
Source: Int Dent J. 2025 Oct 10;75(6):103946. doi: 10.1016/j.identj.2025.103946 (PMC12547215; doi:10.1016/j.identj.2025.103946)
Supplement: Supplementary file 1 [file mmc1.docx]

**SUPPLEMENT TABLES**

**Appendix table 1. Distribution of Indian migrants into homogenous groups based on gender.**

| **Males** | **Females** |
| --- | --- |
| Age (18-35 years; 36-65 years), length of stay in the Netherlands (< 5 years; ≥ 5 years), place of origin in India (name of state and city), education level (low; high) and their occupation in the Netherlands. | Age (18-35 years; 36-65 years), length of stay in the Netherlands (< 5 years; ≥ 5 years), place of origin in India (name of state and city), education level (low; high) and their occupation in the Netherlands. |

**Appendix table 2. Code creation process.**

| Code development | Sources used | Details of methods |
| --- | --- | --- |
| Initial code sources | - Theory - Review of literature - Data from quantitative phase of the project - Identification of initial themes - Initial theme saturation was achieved | Studies the Andersen’s behavioural model.  Also used data from the developed questionnaire, based on this model.  PI and co-I discussed the theory driven themes, and initial definitions and descriptions of each theme was recorded as initial code development.  The discussions exhausted till no new themes could be identified.  A total of five zoom meetings, each lasting between 60-90 minutes took place over a period of two months |
| Initial code development | - Assigned initial codes (open coding) to raw data - Developed more codes from raw data - Intercoder testing was performed | Inductive method  PI and co-I coded one sample transcript together. They assigned line-by-line codes to the data. They discussed the emerging codes, providing preliminary definition and description to each code.  Deductive method  Then sample data was assigned theory driven themes, to see how many emerging codes could be placed under the themes.  A total of three zoom meetings, lasting 50-60 minutes took lace over the next two weeks. |
| Codebook development | - Used six components of coding methods for raw data [x] - Intercoder testing and training performed | Out of six, we used four components of code development, namely code name, definition, description, and a relevant example for each code.  The co-I read the theory and discussion sessions mentioned above helped in reducing her doubts and her increased her familiarity with the codes.  We coded two other transcriptions together, to see if there were any persistent disagreements and also to check how smoothly the coding went.  This process spread over four weeks, in multiple sessions and we spent almost 8-10 hours coding them. |
| Codebook application | - Application of the sample coding to entire dataset - Codebook reviewing - Intercoder testing - Confirmation of coding categories completed | With each transcription, discrepancies in assigning codes reduced.  In addition, PI also invited the co-supervisor to verify the coding criteria for clarity, and uniformity in code definition ad understanding.  A few codes needed to have clearer description. Some codes were clustered and all codes were eventually placed under the relevant themes.  A few new themes also emerged, and after discussion, it was agreed not to use them as they did not directly address the research question.  The codebook was re-organised and we went through all first three transcripts once again with revised codebook.  By the time we finished the last three transcripts, there was total agreement on codes.  This entire process took another three months and additional twenty hours of coding time. |
| Interpretation | - Results presented - Conclusion and recommendations made | Based on the codes assigned under themes, results were presented.  Initial draft of the manuscript/ paper was written and reporting phase began. |

**Appendix table 3. Examples of codes from consensus codebook**

| Code name | Code definition | Code description | Example text |
| --- | --- | --- | --- |
| Definition of a healthy mouth | Perception of people about what they think healthy mouth means to them. | Wherever there is description/ meaning about healthy mouth and how important is it to them. No mouth symptom, no pain, no disease in mouth, no swelling.  Also included will be healthy mouth, like able to eat, smile, and have good looking teeth. | ‘Like when you're eating something that is a bit harder than the normal food. That you are not feeling any kind of pain, or hard to, hard to even break it into pieces’. |
| Means of maintaining a healthy mouth | Methods or ways in which people keep their mouth healthy. The way they take care of their mouth and teeth, in other words-Oral hygiene practices. | Where people talk about what all they use to clean their teeth and mouth. The products they use also include adjuncts, like additional methods of maintaining mouth hygiene. For example, mouthwashes, dental floss, etc.  Also included will be any alternate methods that are unconventional, like traditional methods they may use to clean their teeth. | ‘I do use, uh, yeah, the toothbrush and paste, uh, that's a routine also a tongue cleaner, uh, cause that's also been part of my routine mm-hmm <affirmative> and floss from time to time’. |
| Influence of self-realization | What people consider as internalization process of becoming aware of what is good for them | Where people mention what or who made them aware of what was wrong in their hygiene practices. What made them change their normal way of maintaining oral hygiene and pick up a new habit that they felt was better. | ‘I see my life a bit different. I mean, we are all from India, but typically when we got married, I saw her (wife) dental habits far better than mine. So some, some extended dependent on the family and the upbringing’. |
| Cultural cohesion | Levels of integration or levels of acculturation among the Indian migrants | Where conversations are about how they interact with the Dutch, the problems associated in communication, and understanding the cultural differences.  Also included are the ease or problems while interactions with Dutch neighbours, workplace, shopping, making appointments for health or oral health (with GP’s or dentist).  In addition, what they like or dislike or don’t understand about Dutch culture, especially in relation to oral health or dental setups. | ‘I've done that, that integration course, and I feel quite comfortable. But I cannot also claim to be 100% integrated or kind of Dutch. But I ask myself that, I need to keep my own identity as well. So, you know, as long as I can mix with others, yeah, as long as I can understand that the points of view of others and as long as, you know, I can communicate my point of view, then I'm making progress’.  ‘But I do have, uh, you know, Dutch employees in my team. I mean, it's a very big international, uh, company, but yeah. I mean, there are few Dutch colleagues as well, who are friends who, who I'm, whom you socialize with as well’. |
| Shift in diet | Any change observed in eating patterns since moving to the Netherlands- Influence of migration on food habits | Talk where people feel their food habits, pattens of eating, timings of eating and what they eat has changed since they now live in the Netherlands.  Any conversation on food, drinks, alcohol, or any habit that they either started or left since migration.  Also, what they did in India and how is it different in the Netherlands and why.  Also, if no change, then why so. | ‘There is no defined time of eating in India. I mean, you can always get something, even if it's not prepared on delivery. So for me, munching reduced a lot here’. |
| Changes in oral hygiene practices | Any change observed in maintain oral hygiene routine since moving to the Netherlands- Influence of migration on oral hygiene habits | Conversations about changes felt by people about changes in their hygiene routine. This can be related to change in brushing routine, changes in products used or changes in habits and why.  What reasons are given for changes.  If no change is felt, then why so. | ‘For me it has improved for me the mainly the second brush before the night, it's good for oral hygiene’. |
| Dental experiences in the Netherlands | What sort of experiences Indians have had with the dentist in the Netherlands | All positive or negative experiences, details or incidences related to these experiences. Details about what made them go to a dentist in Netherlands, how was the experience while making appointments, at the reception, waiting area, with the dentist. All that they can remember or want to talk in detail. What they felt and what was their conclusion about these experiences.  Also, if positive, then exactly what they liked and if negative, then also what triggered negative reaction.  How these experiences are different from India. Do they compare? If yes, in what ways. | ‘So, but I (the dentist) do recommend that he said, that you do come and visit me or us, at least twice a year so that you can continue your good health. And let me give me some tips of, you know, dental care, and you know, switching to electric toothbrush and so on. So that was my experience. And now I diligently go twice a year’.  ‘I think it is very clean here. In India, people have a mentality that if someone is in my clinic, I need to get money out of them. Here they don’t think like that. What they have is what they will tell you. I like it here’. |
| Insurances and costs | Do they have dental insurance? If yes, then how if influences dental treatment in the Netherlands. | Talks about what they understand about purchasing dental insurance in the Netherlands. Since it is an additional insurance, do they buy it? If yes, why? How dental insurance influences their dental visits.  How do they feel about the costs of dental treatment in Netherlands.  If they don’t have dental insurance, why not. What conversations evolve around insurances and costs of dental treatment. | ‘So when I first went to the dentist over here, it was not due to any problem I had. It was a, you know, when, when you do your medical insurance here, dental policy is part of the package as an option. And then you, you kind of try to figure out, and then you say, you figured out that, you know, you are allowed to go to a dentist twice a year, just for checkups. I said, ok, lets go…let's go and find out’. |
| Dentist-patient relationship | Rapport between the dentist and themselves. How do they perceive their interactions with the dentist. | All conversations that highlight what Indians feel when they talk to the dentist in the Netherlands. Talks that revolve around- can they communicate their problems? Do they feel they can discuss their issues with the dentist freely? How comfortable they are with the dentist? Do they feel confident in the advice of the dentist?  Also, do they compare their interactions with the dentist in the Netherlands to India? What positives and/or negative points emerge. | ‘At some point I feel like they're not seeing the real pain from the patient's perspective. They're just going beyond the formula or whatever it is, the steps they have it in three different. That's what I feel’.  ‘I said earlier…you don’t get what you are looking for, so satisfaction is not there. But going to a clinic and get appointments, etc are not that difficult. But in case the dentist is not communicating well with you then what will you do with appointments’. |
| Treatment procedures | Perception regarding treatment procedure carried out by the dentist in the Netherlands. | Talks that circle around their reason for visiting a dentist in the Netherlands. What was their problem and what kind of treatment was offered and what did they do. Were they satisfied with the treatment or not. Discussions around time taken for the treatment (duration), their satisfaction with the treatment, number of times they had to go for the treatment, etc.  Are there any comparisons made in treatments between Netherlands and India? | ‘I think they don’t have much experience though. They try what they can…they are never rude….but they don’t know much’.  ‘I think…every time I went to a dentist here, I went due to pain and every time they took my teeth out. It has happened 2-3 times now. I went and each time they took out two teeth…in total they have now removed 6 teeth. So now I don’t go anymore…because I went to India and got my treatment done…because here they prolong the treatment so much and they don’t even tell you clearly…We asked them, and they told us it will be a 3 year long treatment. This and that’. |
| Perception regarding dental visits | The perception among Indians about visiting a dentist in general. | Talks about what Indians feel about visiting a dentist in general. Have they ever visited a dental clinic, be it in Indi or in Netherlands? If they do, for what reason do they usually go to a dentist. If not, then why so.  Have they stared going to a dentist in Netherlands and if so, why? What makes them go to a dentist now. Conversations about reasons to visit or not, their beliefs and thoughts regarding this concept of dental visits. | ‘I mean, uh, to be honest in India, uh, I visited the dentist the first time I had a cavity. Okay. And that I needed an RCT. But, uh, otherwise, uh, no, until, and unless there is no pain. Yeah. There is no need. There is no need to go to the dentist’.  ‘It is also in our insurance that we go do a dentist and if you don’t go then mostly, they call you every three months or six months as a reminder’. |
| Practice management | Perception about the overall environment in the Netherlands that is related to oral healthcare use. Like the laws, dental clinics, etc | Apart from the immediate environment talked already, what is the role of workplace in encouraging Indians to visit a |  |
| Changes in oral healthcare utilization |  |  |  |
|  |  |  |  |

**Appendix table 4. Examples of codes, sub-categories, categories, and a theme from analysis**

| Meaning unit  examples | Condensed meaning  description close to text | Condensed meaning  sub category | Category / theme | Theory |
| --- | --- | --- | --- | --- |
| ‘And in the first two, three years, i, i… was not taking dental insurance, but then it came to the company…I had the insurance, but i was not taking it. but once i had these incidents (dental problems) that i had to go to dentist because of critical situation, then i realized, oh, this much it's all covered in, in the, the insurance. So, for me initially, the perception was the cost, but then i realized, just take the insurance, which the company gives for free anyway. it, so cost is not the issue. it's, it's more my tendency to go there’. | Indians do not see cost as a reason for not visiting a dentist, especially when they realise what is covered by dental insurance. | Cost no issue | Insurances and costs | Enabling resource |
| ‘They (Dutch dentist) did not communicate well. And the, the way she (dentist in the Netherlands) told me that, okay, this has to be done (root canal treatment). This has to be, then this is the, she did not properly explain the repercussions that, okay…... And I mean, if I, I think if as a, as a patient or as a client, if I have no pain whatsoever before going there and she does a job and then as it starts paining, like anything, and I, I can't even sleep then I, I don't need that thing. Right. Yeah’. | Indian migrant expresses dissatisfaction in the way Dutch dentists deal with them. She goes on to explain that treatment plan was not discussed to her satisfaction. | Dentists do not communicate well | Dentist patient relation |  |
| ‘Uh, but i expected a bit more like, uh, when the, the next one I had over here, the root canal, uh, I thought, uh, by comparing my, uh, Indian doctor did a better job because, um, yeah, <laugh> when the doctor (in Netherlands) was drilling, uh, he saying, yeah, I can't find the nerve that I need to empty. I was like, what are you saying? He… are you having a drill in your hand? and you're saying, I am having difficulty. maybe we do it again. I was like, no, just fix it means you have the teeth open, you have to fix it. And they were like, yeah, let's see. and then finally he did it. He did the stuffing and then everything. But, uh, yeah, it was a bit scary’. | Indian migrant describes his dental experience with the Dutch dentist. He talks about the way his treatment was handled by the Dutch dentist that was not comfortable for the Indian migrant. | Trust issues with treatment procedures | Treatment procedures |  |
|  |  |  |  |  |


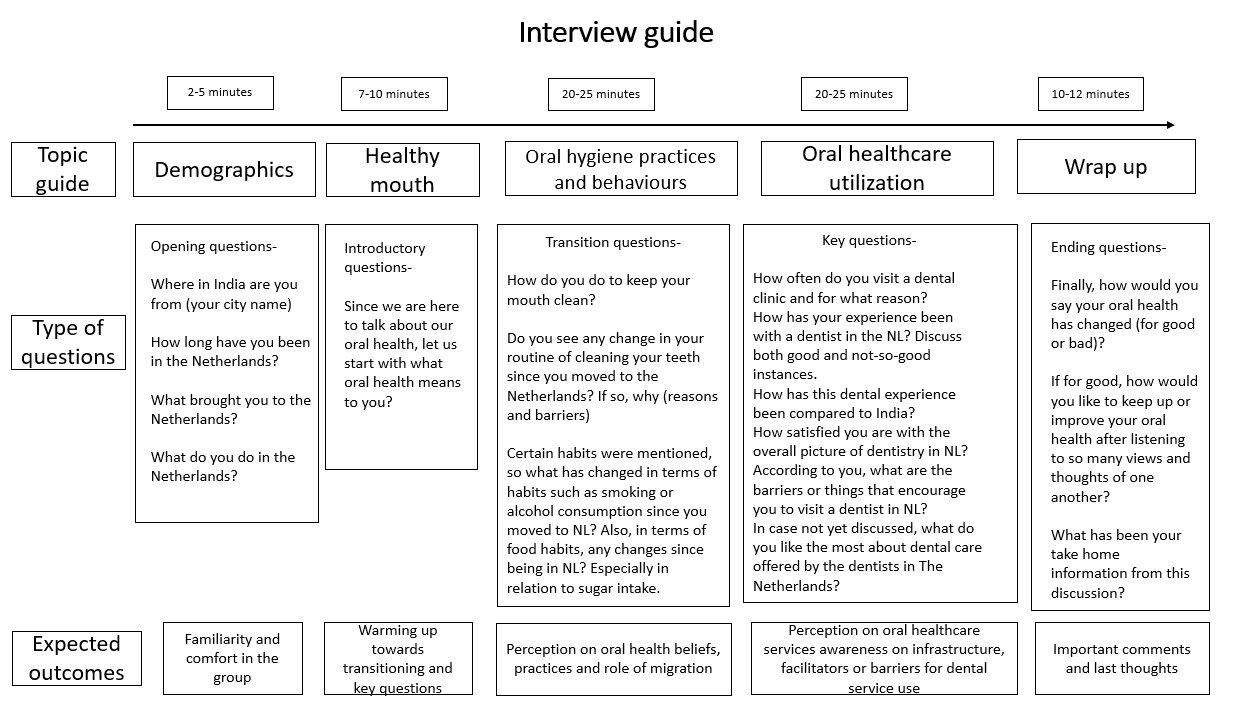


**Appendix figure 1: Interview guide map based on Andersen’s (amended) behavioural Model (1995)**
